# Treatment of Mandible Fractures Using a Miniplate System: A Retrospective Analysis

**DOI:** 10.3390/jcm9092922

**Published:** 2020-09-10

**Authors:** Lauren Bohner, Fabian Beiglboeck, Stephanie Schwipper, Rômulo Maciel Lustosa, Carla Pieirna Marino Segura, Johannes Kleinheinz, Susanne Jung

**Affiliations:** 1 Department of Oral and Maxillofacial Surgery, University Hospital Münster, 48149 Münster, Germany; fabian.beiglboeck@ukmuenster.de (F.B); stephanie.schwipper@gmx.net (S.S.); johannes.kleinheinz@ukmuenster.de (J.K.); susanne.jung@ukmuenster.de (S.J.); 2 Department of Dentistry, State University of Maringá, 87020-900 Maringá, Brazil; romulo.lustosa@gmail.com; 3 Department of Head and Neck Surgery Daniel Alcides Carrion National Hospital, 07016 Bellavista, Peru; cmarino89@gmail.com

**Keywords:** mandibular fracture, jaw fracture, maxillofacial injuries

## Abstract

Three-dimensional (3D) mini plate systems are used in the treatment of mandibular fractures. The system is advantageous in comparison to conventional plates due to the stabilization of tension and compression areas, improved initial stability, and biomechanical behavior. The aim of this retrospective study was to evaluate the use of a 3D miniplate system for the treatment of patients with mandibular fractures. Patients with mandibular fractures treated with a 3D plate system at the Department of Oral and Maxillofacial Surgery, University Hospital Münster, during a period of 5 years, were included in this study. Mandibular fracture conditions and minor and major post-operative complications were reported. In total, 336 patients and 391 mandibular fractures were assessed. The most common fracture site was anterior mandible, and 155 cases involved a tooth-bearing area. Minor complications were seen in 8.03% of cases, whereas only 1.49% of patients suffered from major complications. The treatment of mandible fractures using 3D miniplates resulted in fracture reduction with a low complication rate.

## 1. Introduction

In the management of mandible fractures, it is essential to restore occlusion, temporomandibular function, and facial esthetics [1,2]. Treatments range from non-surgical management to surgical procedures with osteosynthesis plates, according to the fracture complexity [3]. In cases where surgical procedure is required, internal rigid fixation techniques promote better stabilization and consolidation of fractured bone. Nevertheless, a successful treatment is influenced by various factors, such as the location and complexity of the fracture, and the plate system and its geometry [4]. Infection, paresthesia, and malocclusions are reported as possible post-operative complications after osteosynthesis of mandible fractures [3,4,5,6].

To ensure successful osteosynthesis with minimal invasive approach, three-dimensional (3D) miniplate systems can be used [7,8,9,10,11]. The system comprises two miniplates joined by interconnecting crossbars and fixed to the bone with monocortical screws [9,10]. When placed in the fracture line, the 3D miniplate system ensures fracture stabilization, regardless of plate thickness and geometry [10]. The placement of screws in a square pattern on either side of the fracture creates a solid platform that increases resistance to twisting and rotation of the longitudinal axis of the plate [10].

Simultaneous stabilization of tension and compression areas, improved initial stability, and biomechanical behavior are the main advantages of the 3D miniplate system in comparison to conventional plates [9,11]. In addition, this system can be easily operated by the patient due to its shape and low profile, which enables reduced palpability and contouring. Compared to the conventional system, previous studies using 3D miniplates showed a reduction in 58% of post-operative complications [12,13]. However, few studies reported the clinical efficacy of 3D miniplate systems for treating mandibular fractures. The aim of this retrospective study was to evaluate the use of the 3D miniplate system for the treatment of patients with mandibular fractures.

## 2. Experimental Section

Patients who had attended the Department of Oral and Maxillofacial Surgery at the University Hospital Münster over a period of five years were included in this study. Inclusion criteria were the presence of mandibular fractures treated with a 3D miniplate system (Trauma 2.0, Medartis, Basel, Switzerland). The system is adapted for craniofacial fractures and ensures the semi-rigid fixation of fractured bone. Exclusion criteria consisted of the use of alternative techniques, condylar fracture, or minimal fractures treated with conservative approaches.

### 2.1. Pre- and Intra-Operative Data Assessment

Patient data (gender, age, weight, medical history), surgical protocol, and radiographic exams were recorded. Fracture assessment was performed with panoramic radiograph, occlusal, and Clementschitsch view projections.

Based on pre-operative panoramic radiographs, the localization of jaw fractures was classified as follows: (1) right and left mandibular angles, (2) right and left mandible body, (3) anterior mandible. The degree of displacement was classified as either major or minor. The presence of teeth at the fracture line, dental trauma, and additional soft-tissue injuries, as well as cranial, thoracic, and abdominal traumas were also assessed. In addition, the operation time was recorded.

### 2.2. Surgical Procedure

**Figure 1:**
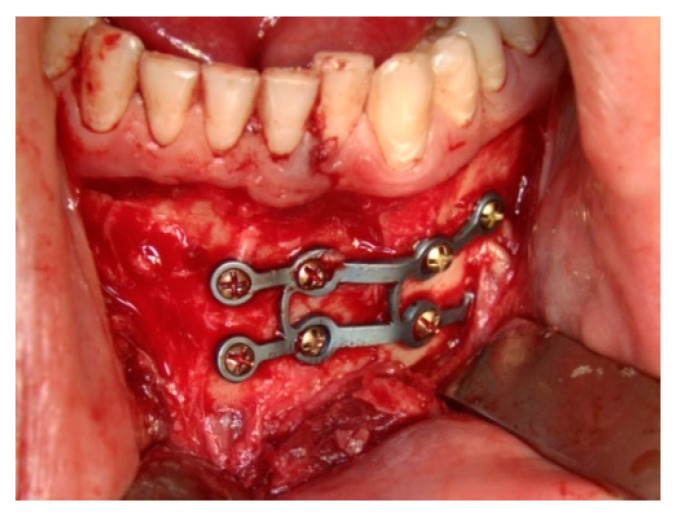
Internal fixation of the anterior fracture using the plate M-4484 (Medartis Trauma 2.0).

Surgical protocol was followed as briefly described: In order to access bone, an intra-oral vestibular incision was made and the mucoperiosteal flap was raised. After reducing bone fragments into their anatomical position, intermediate intermaxillary fixation (IMF) was performed using wire ligature or IMF screws to ensure adequate occlusion. Fracture osteosynthesis of the fracture was performed using the 3D locking miniplate system (Trauma 2.0, Medartis, Basel, Switzerland). Plates were adapted to the bone anatomy and fixed to the cortical bone using monocortical 2.0 mm screws (Figure 1). After IMF removal and proof of occlusion, incision closure was performed with 3.0 Vycril sutures. When necessary, a postoperative IMF was applied to stabilize the occlusion using wire ligature, IMF screws, or head-chin bandages. Patients were hospitalized for 7 to 14 days. During this period, patients received a liquid diet or enteral nutrition, and a soft diet was recommended for the first 30 days after the procedure. Analgesic was prescribed in case of pain, and antibiotic therapy (Amoxiclav 875/125 mg; in case of penicillin allergy: Clindamicin 600 mg) was prescribed for a period of 5–7 days to prevent bacterial infection.

### 2.3. Post-Operative Data Assessment

Treatment protocol, presence of multiple fractures, and the need for additional plate systems were assessed. Additionally, gaps between fractured segments and the presence of minor and major post-operative complications were recorded. Post-operative pain was assessed using the Visual Analogue Scale (VAS). Categories of pain were defined as follows: 1–3 = mild pain, 4–6 = moderate pain, and 7–10 = severe pain.

## 3. Results

### 3.1. Pre- and Intra-Operative Data Assessment

Over five years, 582 cases of mandible fractures were surgically treated. Of these, 336 patients received a 3D miniplate (Trauma 2.0, Medartis, Basel, Switzerland). and were included in this study. Fifty-five patients showed a second fracture, totalizing 391 treated mandibular fractures.

Participants consisted of male (73.5%) and female (26.5%) patients. At the time of the surgical osteosynthesis, most patients (46.72%) were between 21–40 years old. Among 70 patients that presented a comorbidity (e.g., hypertension), 31.9% presented cardiovascular disease and a total of 22 patients required medication (Table 1).

### 3.2. Fracture Assessment

The main fracture characteristics are reported in Table 2. Brutality acts (38.7%) and accidents (35.41%) were the main causes of fractures. The most common fracture sites were the anterior mandible, mandibular angle, and mandible body. In addition to these conditions, 290 patients presented a second mandible fracture. Additionally, 37 patients presented with other facial fractures, of which 11 (11%) had zygomatic fractures. Most fractures (86.4%) involved the dental area, and in 155 cases, fractures were located in the tooth-bearing area. Furthermore, 36 patients reported a recent third molar extraction on the fracture site. Dental trauma occurred in 93 cases, as follows: 33 enamel-dentin fracture, 11 avulsion teeth, 7 subluxation teeth and 5 horizontal fractures.

**Figure 2:**
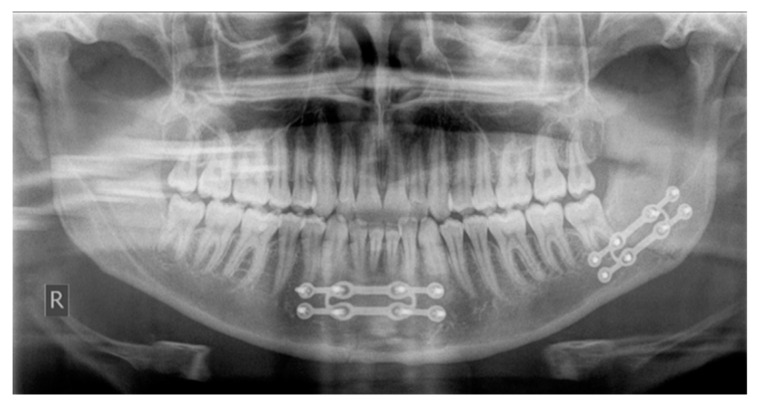
Double mandibular fracture treated with two Grid-Plates (Modus Trauma 2.0, Medartis).

In addition, 107 patients received intermaxillary fixation to treat condylar fractures, and seven patients were treated with a head-chin bandage. Postoperative antibiotic therapy was prescribed for 320 patients (95.2%). Figure 2 shows the post-operative panoramic radiograph of a double mandibular fracture treated with 3D miniplates. In 314 patients, the plate was removed after four months, whereas for six patients, the second surgical procedure occurred in two months. The mean operation time (standard deviation) was 80 (60.72) min.

### 3.3. Post-Operative Data Assessment

In 87.79% of cases, there was a minor displacement of bone fragments, and the gap between fractured sites was less than 1 mm. Furthermore, in 89.3% of cases, no displacement was seen in the panoramic radiography.

Post-operative minor complications were observed in 8.03% of the patients, which were treated with antibiotic therapy and local procedures. Only 1.48% of the patients presented major complications (Table 3). No plate fracture was reported, and no plate was lost due to complications, although screw loosening occurred in 1.78% of cases. Due to infectious or technical complications, the plate had to be replaced in five patients (1.5%).

Eighty patients experienced no pain (23.8%), whereas 150 suffered only mild pain. Moderate and severe pain were reported by 96 (28.6%) and eight patients (2.4%), respectively.

## 4. Discussion

The present study shows a retrospective assessment of mandibular fractures treated with 3D miniplates. According to our results, post-operative complications occurred in 8.03% of cases, most of which were treated by antibiotic therapy and local management. This finding is consistent with previous studies evaluating 3D miniplates, in which the percentage of complications reached 6%.

Sensibility disturbance was reported by a high number of patients. This may be related to the nerve injury during bone manipulation and plating, since only few of them reported sensibility disturbance after plate removal. Moreover, wound dehiscence, which tends to occur when the plate is placed close to the incision [10,14], was the most prevalent post-operative complication among patients observed in this study. Out of the 336 patients assessed, only five of them presented major complications and underwent a second surgery to replace the titanium plates. The low rate of major complications is consistent with the findings of Singh et al. (2016) [10], who reported no complications in 20 patients treated with either locking or non-locking 3D plates.

Currently, the treatment of mandibular fractures is founded on two principles. The first one claims that the plate must provide sufficient rigidity to avoid displacement between fragments during functional movements. This is achieved by a rigid fixation using solid plates [15,16]. Conversely, the Champy technique suggests the use of semi-rigid fixation, on which tensional forces are neutralized when miniplates are placed according to the ideal line of osteosynthesis [17,18].

Three-dimensional miniplates were introduced as a modification of Champy´s method, on which two plates are connected by interconnecting crossbars, and separated by a distance of 4–5 mm from each other. Due to their malleable format, they can be anatomically adapted without distortion. Thus, tensile and compressive forces are simultaneously neutralized, providing bone alignment and undisturbed healing. In addition, due to their shape and low profile, these plates allow reduced palpability and contouring in the fractured jaw area, decreasing the risk of paresthesia [19].

A recent meta-analysis showed that 3D and conventional miniplates present a similar complication rate [18]. In regard to post-operative infection, wound dehiscence and paresthesia, both methods provided similar results [18]. Conversely, post-operative complications, such as mal-occlusion and trismus, appear to occur more frequently when fractures are fixed using conventional miniplates [9,20]. These findings are in agreement with Mohd et al. (2019) [21], who showed a lower mobility and improved occlusion restoration for parasymphisis fractures treated with 3D miniplates. Reduced rates of occlusion-related complications may be associated with improved plate stability. However, as a comparison among different plate systems was beyond the scope of this study, no conclusion regarding the best fixation method can be drawn from these results.

Another advantage of miniplates is the easy handling, which contributes to the shortening of intra-operative time. The present study shows an acceptable mean operation time, which ranges from 12 to 630 min, according to the clinical condition and complexity of the case. Moreover, the treatment of anterior mandibular fracture using 3D miniplates reduced operative time [22]. This might be related to the capability of achieving a stable condition using 3D miniplates, as well as the simultaneous stabilization of tension and compression zones provided by the plate. Nonetheless, a limitation of this system is the need to remove the entire plate when a failure occurs. In this case, two separate plates allow an easier management of plate failure.

In this study, most of the fractures treated with a 3D miniplate system showed a gap of less than 1 mm and no displacement between bone fragments. These factors play an important role in the final outcome, since one of the main purposes of the mandibular fracture treatment is to provide fixation and stabilization of the fractured bone [16]. The optimal results are possibly related to the fact that only few cases showed unfavorable displacement of bone segments.

A previous systematic review showed a success rate of 88.9% for treatment of mandibular fractures with 3D miniplates. This result relies on the area of the fracture zone and fractured bone displacement. Regarding mandibular angle fractures, lower success rates have been reported [23]. In this study, a large number of fractures were located at the mandibular angle, which might be considered unfavorable due to the strong displacement caused by muscular movements [23,24]. This confirms previous findings in the literature, which classify mandibular symphysis and mandibular angle as the most fractured sites [3].

In regard to the patients’ data, our study provides further evidence for the higher prevalence of male patients as a result of brutality acts or accidents. In 155 cases, the fracture occurred in the tooth-bearing area, although only five required tooth removal. Further extractions involved dental trauma indications. According to the literature, indications for tooth extraction are related to the teeth’s condition, and the impossibility of reducing and fixating the fracture. Whereas fractured teeth, periodontal diseases, or caries are conditions that may require tooth extraction, the complication rate does not increase if a healthy tooth is maintained [25].

Several treatment- and patient-related aspects can lead to complications, such as patient medical conditions, surgical managements, and patient compliance. These factors should be considered individually to provide a true relation between post-operative complications and the plate system. A limitation of the study is the bias related to the study design, since the choice to evaluate a 3D miniplate system for surgical treatment of mandibular fractures introduces selection bias. Furthermore, measurement of displacement is restricted to bidimensional images. For further studies, the authors recommend a 3D assessment of plating to obtain accurate results. Randomized trials comparing different plate systems and taking into consideration the case complexity should be performed to indicate the appropriate plate system according to individual situations.

## 5. Conclusions

Within the limitations of this study, the treatment of mandible fractures using 3D miniplates resulted in fracture reduction with a low complication rate and an adequate surgical procedure time.

## Figures and Tables

**Table 1:** Patient´s data.

Patient’s Data		Number of Patients (*n*)
Gender		
	Male	247
	Female	89
Age		
	11–20	102
	21–40	157
	41–60	61
	>60	16
Co-morbidities		
	Cardiovascular disease or coagulation alterations	49
	Respiratory diseases	31
	Infectious disease	8
	Diabetes mellitus, Osteoporosis or Tumor	5
Medication		
	Thyroid hormones or symphatomimetic drugs	10
	Angiotensinconverting-enzyme (ACE) inhibitors	07
	Acetylsalicylic acid (ASS) and Beta-Blocker drugs	06
	Anti-depressant and anti-epileptics	4
	Insulin	3
	Benzodiazepines, opioids or coumarin anticoagulant therapy	2

**Table 2:** Fracture characteristics.

Mandibular Fractures		Number of Patients (*n*)
Localization		
	Anterior mandible	159
	Mandibular angle	129
	Mandibular body	108
Mandibular displacement		
	None	83
	Weak	152
	Moderate	118
	Strong	38

**Table 3:** Minor and major post-operative complications.

Post-Operative Complications		Number of Patients (*n*)
Minor complications		
	Infections	Total: 10
	- Abscess	6
	- Fistula	2
	- Local infiltration	1
	- Osteomyelitis	1
	Dehiscence or healing complications	26
	Plate exposition	2
	Sensibility disturbance	Total: 156
	- Before plate removal	156
	- After plate removal	22
	Screw loosening	6
Major complications		
	Pseudoarthrosis	2
	New platting	5
